# High temperatures increase the virulence of *Vibrio* bacteria towards their coral host and competing bacteria via type VI secretion systems

**DOI:** 10.1371/journal.pbio.3002788

**Published:** 2024-09-04

**Authors:** Weiquan Wang, Kaihao Tang, Xiaoxue Wang

**Affiliations:** 1 Key Laboratory of Tropical Marine Bio-resources and Ecology, South China Sea Institute of Oceanology, Chinese Academy of Sciences, Guangzhou, China; 2 University of Chinese Academy of Sciences, Beijing, China

## Abstract

The bacterial pathogen Vibrio coralliilyticus induces severe coral diseases in warming oceans. This Primer explores a PLOS Biology study revealing that high temperatures activate two type VI secretion systems in V. coralliilyticus, enhancing pathogenicity by deploying toxic effectors against competing bacteria and coral cells.

## Teaser

The bacterial pathogen *Vibrio coralliilyticus* induces severe coral diseases in warming oceans. A study in *PLOS Biology* reveals that high temperatures activate 2 type VI secretion systems in *V*. *coralliilyticus*, enhancing pathogenicity by deploying toxic effectors against competing bacteria and coral cells.

**Fig 1:**
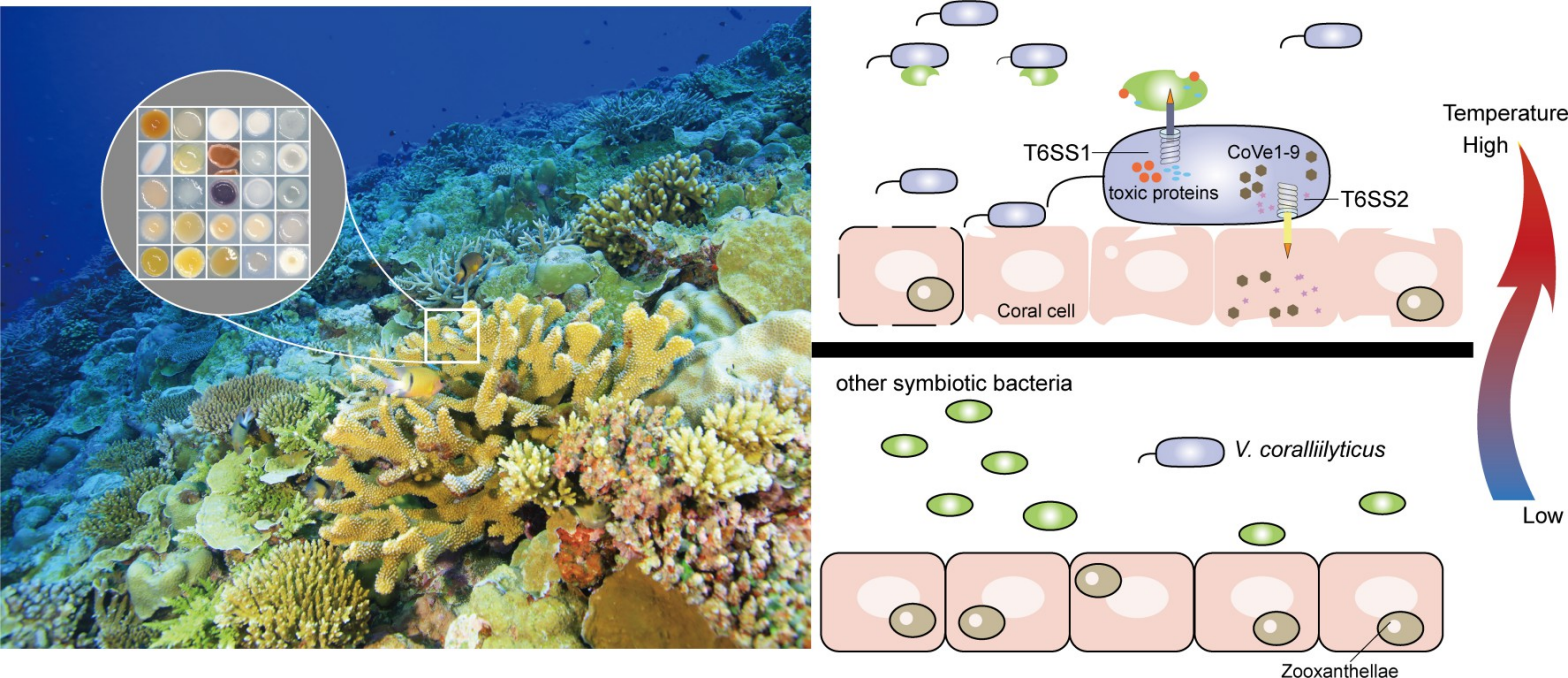
Schematic depicting interactions between coral pathogen, cohabiting bacteria, and coral host. Activation of 2 T6SS systems at elevated ocean temperatures enhances *V*. *coralliilyticus* pathogenic potential by secreting antibacterial effectors to kill bacterial competitors and secreting anti-eukaryotic effectors.

Rising sea temperatures and heat waves pose significant threats to coral reefs worldwide. Pathogens belonging to the *Vibrio* genus are particularly concerning due to their association with temperature-related diseases, which exhibit peak infection rates in both humans [1] and corals [2]. Increased summer temperatures correlate with heightened outbreaks of *Vibrio cholerae*, the cholera pathogen, highlighting the direct influence of temperature on *Vibrio* pathogenicity [3], although specific mechanisms of temperature-related infections remain understudied. *Vibrio coralliilyticus*, an opportunistic coral pathogen sensitive to temperature fluctuations, infects multiple coral species and poses a global threat to reef ecosystems, especially when temperatures exceed 27°C [4]. Despite coral hosts having multiple defense mechanisms, bacteria like *V*. *coralliilyticus* have evolved diverse strategies for colonization and invasion. Previous studies have explored these strategies, including the secretion of proteases and hemolysins, modulation of motility, and competition with commensal bacteria via prophage induction [2,5]. In the study published in the current issue of *PLOS Biology*, Mass and colleagues revealed the activation of 2 type VI secretion systems (T6SS) in *V*. *coralliilyticus* under high temperature [6]. They identified a range of antibacterial effectors discharged by T6SS1 and anti-eukaryotic effectors deployed by T6SS2 (Fig 1), enabling it to circumvent coral host’s defense mechanisms. The finding reinforces the versatile strategies employed by the coral pathogen to invade and infect corals.

The coral microbiome plays a critical role in maintaining coral health. Coral animals establish commensal relationships with photosynthetic endosymbiotic dinoflagellates and a diverse array of microbes, encompassing bacteria, fungi, archaea, and phages. The concept of the “coral holobiont,” introduced by Rosenberg, integrates the coral organism and its commensal microbes into a cohesive functional unit essential for adaptation and evolution [7]. This concept underscores the significance of interactions between the coral host and its microbial communities in shaping the resilience and health of coral reefs. Commensal bacteria within this microbiome function as a primary defense line against pathogens, complementing the coral’s own immune system.

T6SSs are specialized transport apparatuses capable of targeting both eukaryotic and prokaryotic cells, playing a crucial role in microbe–microbe and microbe–host interactions [8]. Structurally akin to the contractile bacteriophage tail, T6SS is among the largest dynamic assemblies found in gram-negative bacteria [9]. Mass and colleagues conducted a comprehensive analysis of T6SS in the pan-genome of *V*. *coralliilyticus*, uncovering 2 conserved T6SSs across all genomes. Their study focused on elucidating the regulation and function of these systems, utilizing western blotting to monitor the expression and secretion of key T6SS structural components, namely, VgrG1 and Hcp2. Their findings highlighted the temperature-dependent activation of both systems, with peak activity observed in nutrient-rich conditions at 28°C. The two T6SS systems seem to be subject to differential regulation when temperature and nutrient level changes, yet the regulatory network governing both T6SS systems remains unclear. Nonetheless, the conditional activation of these secretion systems by environmental factors such as temperature and nutrient levels underscores the intricate link between climate change and microbial competitive fitness, affecting community dynamics and their pathogenic potential. A similar observation has been made for the T6SS in *V*. *cholerae* whose activities are also modulated by environmental factors that relate to oceanic changes [10]. Such regulation suggests that rising ocean temperatures could further exacerbate the virulence of pathogens like *V*. *coralliilyticus*, reshaping coral reef ecosystems in profound ways.

Colonization at the appropriate site is the initial step in bacterial infection. Coral commensals employ various mechanisms to hinder pathogen colonization, such as disrupting signaling pathways, producing antibiotics, and inhibiting pathogen-encoded glycosidases. Conversely, coral pathogens utilize diverse strategies to outcompete resident bacteria for successful colonization. In interspecies competition assays, the researchers demonstrated that T6SS1 in *V*. *coralliilyticus* delivers antibacterial effectors into neighboring commensal bacterial cells, effectively eliminating them. These effectors are antagonized by cognate immunity proteins within the pathogen to prevent self-intoxication. Similar mechanisms are observed in *V*. *cholerae*, where T6SS contributes to the elimination of commensal bacteria [8].

Although the T6SS is related to bacteriophages that do not target eukaryotes, it has become evident that many T6SSs can translocate anti-eukaryotic effectors. The role of T6SS2 in anti-eukaryotic activities was investigated using *Artemia salina* as a model for lethal/survival experiments. Findings showed that disabling T6SS2 significantly reduced lethality, whereas T6SS1 inactivation had minimal effect. Comparative proteomics analyses identified the effector proteins responsible for anti-eukaryotic actions of T6SS2. Notably, these effectors were toxic to yeast but not *E*. *coli*, with 9 novel anti-eukaryotic effectors identified. Pathogens can employ various effectors to alter and control various cellular processes in eukaryotic cells, enabling them to evade the host’s immune system. Considering that *V*. *coralliilyticus* can infect diverse coral species, future research could explore whether T6SS2 effectors function across different coral species and their interactions with host immune systems. Investigating whether coral genomes encode immunity proteins to counteract these toxins would be valuable. This study suggests potential strategies for coral reef protection, such as developing coral probiotics that deactivate the pathogen’s T6SS apparatus or neutralize its toxic effectors.

The presence of dual T6SSs enables *V*. *coralliilyticus* to pursue a multifaceted approach—directly attacking hosts while outcompeting bacterial rivals, thus securing its niche within the microbial consortium. One intriguing question is why and how *V*. *coralliilyticus* acquired 2 T6SS systems, which are powerful weapons for survival but also macromolecular complexes costly to maintain. These systems may have been tailored within *V*. *coralliilyticus* over time to meet the specific challenges posed by the coral microbiome environment. Further in vivo research within the coral ecosystem is essential to determine whether both systems are necessary for infecting corals, or if one plays a primary role in the infection process under varying conditions.

While the specific involvement of these antibacterial and anti-eukaryotic effectors in coral disease occurrence and progression remains unexplored, the study underscores the dual role of T6SS systems in enabling a coral pathogen to bypass multiple defense mechanisms of corals. Understanding these mechanisms could inform strategies to mitigate coral diseases and preserve reef ecosystems in the face of rising ocean temperatures and environmental stressors.
